# Lower Systolic Blood Pressure in Normotensive Subjects is Related to Better Autonomic Recovery Following Exercise

**DOI:** 10.1038/s41598-020-58031-5

**Published:** 2020-01-22

**Authors:** Letícia Santana de Oliveira, Anne Michelli G. G. Fontes, Ana Laura Ricci Vitor, Franciele M. Vanderlei, David M. Garner, Vitor E. Valenti

**Affiliations:** 1 Autonomic Nervous System Center, Post-Graduate Program in Physical Therapy, São Paulo State University, UNESP, Presidente Prudente, SP Brazil; 2 Cardiorespiratory Research Group, Department of Biological and Medical Sciences, Faculty of Health and Life Sciences, Oxford Brookes University, Headington Campus, Gipsy Lane, Oxford, OX3 0BP United Kingdom; 3 Autonomic Nervous System Center (CESNA), São Paulo State University, UNESP, Marilia, SP Brazil

**Keywords:** Physiology, Cardiology

## Abstract

Blood pressure (BP) is a cardiovascular parameter applied to detect cardiovascular risk. Recently, the pre-hypertension state has received greater consideration for prevention strategies. We evaluated autonomic and cardiorespiratory recovery following aerobic exercise in normotensive individuals with different systolic BP (SBP) values. We investigated 30 healthy men aged 18 to 30 years divided into groups according to systolic BP (SBP): G1 (n = 16), resting SBP <110 mmHg and G2 (n = 14), resting SBP between 120–110 mmHg. The groups endured 15 minutes seated at rest, followed by a submaximal aerobic exercise on a treadmill and then remaining seated for 60 minutes also at rest, during recovery from the exercise. Cardiorespiratory parameters and heart rate (HR) variability (HRV) (rMSSD, SD1, HF [ms^2^]) were evaluated before and during recovery from exercise. G2 displayed slower return of SBP, rMSSD and SD1 HRV indices during recovery from exercise compared to G1. In conclusion, normotensive subjects with higher resting SBP (110 to 120 mmHg) offered delayed autonomic recovery following moderate exercise. We suggest that this group may be less physiologically optimized leading to cardiac risks.

## Introduction

Cardiovascular diseases are the foremost cause of mortality worldwide^1^. Studies in humans and animals have demonstrated that autonomic dysfunction is closely related to cardiovascular diseases, since autonomic dysfunction implies an inferior prognosis for individuals, increasing the risk of cardiac arrest, infarctions and sudden death^2–4^. Hence, a non-invasive method that evaluates autonomic modulation is heart rate (HR) variability (HRV). It defines the oscillations between consecutive inter-beat intervals (IBI) that are related to the influence of the autonomic nervous system on the sinus node of the heart^5^.

HRV is reduced in hypertension^6^, which is a significant risk factor for the development of cardiovascular disorders and the chief reason of premature death^1,7^. Approximately, one billion people globally are hypertensive and more vulnerable to stroke and sudden death^1,7^. So, due to the hazardous control of blood pressure (BP) levels in the hypertensive population, there is an increase in cardiovascular morbidity and mortality^8^.

According to the most recent guidelines for the management of hypertension, BP is classified as normal for systolic BP (SBP) <120 mmHg and diastolic BP (DBP) <80 mmHg); pre-hypertension for SBP between 129 and 139 mmHg and/or DBP between 80 and 89 mmHg; stage 1 hypertension for SBP between 140 and 159 mmHg and/or DBP between 90 and 99 mmHg and; stage 2 hypertension for SBP values ≥160 mmHg and/or DBP ≥100 mmHg^9^.

Pre-hypertension was formerly indicated to be a risk factor for cardiovascular disease^10^. Guo *et al*.^10^ commenced a systematic review and meta-analysis of prospective studies to evaluate the connection between pre-hypertension and cardiovascular disorders. The authors considered pre-hypertension or high normal BP as baseline exposure, fatal or non-fatal incident stroke, myocardial infarct, chronic heart disease and wide-ranging cardiovascular events as an outcome. As a main assumption, the authors stated that pre-hypertension was associated with increased risk of myocardial infarct, stroke and cardiovascular diseases.

Thus, a non-invasive and practical clinical technique to examine cardiovascular health is the assessment of post-exercise cardiac autonomic recovery^11^. At the start of aerobic exercise, parasympathetic withdrawal primarily increases HR and the subsequent sympathetic activity increases HR and BP. After the end of exercise, vagal reactivation and sympathetic withdrawal promote that HR returns to baseline levels^12^. The scientific research literature maintains that delayed HR autonomic recovery from exercise is related to elevated risks of cardiovascular diseases^13,14^.

Together, they raise the following questions: Is normal BP close to pre-hypertension a possible risk factor? Would normotensive subjects with higher SBP (≈120 mmHg) present slower autonomic recovery following exercise compared to normotensive individuals with lower SBP (<110 mmHg)? In order to attempt to explain this cross-examination, we evaluated autonomic and cardiorespiratory recovery following a submaximal aerobic exercise in normotensive subjects with different SBP values.

## Method

### Strobe guidelines

Our investigation is in accordance with the STROBE (STrengthening the Reporting of OBservational studies in Epidemiology) guidelines. Our study provides comprehensive information concerning the study design, setting, variables, participants, measurements, data sources, quantitative variables description, description of potential sources of bias and statistical methods.

### Population study and eligibility criteria

**Figure 1:**
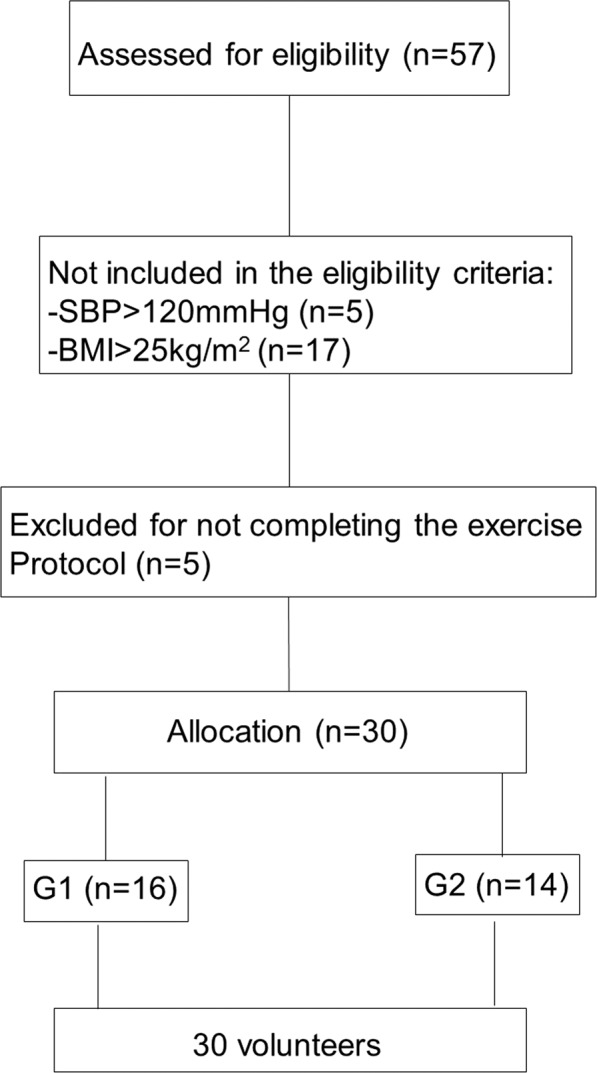
Flowchart.

Initially, we evaluated 57 healthy, physically active college educated males. After excluding subjects owing to higher resting SBP (>120 mmHg), body mass index (BMI) >25 kg/m^2^ and by not completing all necessary stages of the experimental protocol; the final sample was 30 subjects (Fig. 1). Subjects were excluded under the following criteria: overweightness, then cardiorespiratory, neurological, musculoskeletal, renal, metabolic, endocrine and other known or reported deficiencies that prevented the performance of the stated protocols. We omitted subjects under pharmacological treatments, smokers, sedentary and inadequately active individuals consistent with the International Physical Activity Questionnaire (IPAQ)^15^.

The subjects were split into two groups, according to resting SBP: (G1) SBP <110 mmHg, n = 16 and; (G2) SBP between 110 and 120 mmHg, n = 14. This separation was based on the pre-hypertension classification^16^.

### Ethical approval and informed consent

All procedures were evaluated and approved by the Research Ethics Committee in Research of UNESP/Marilia (Number 5406). All experimental protocols were performed in accordance with the 466/2012 resolution of the National Health Council of December 12^th^ 2012. All participants signed a confidential informed consent letter.

### Study design and setting

This is a prospective, observational and analytical study finished at the Autonomic Nervous System Center, UNESP, Marilia, SP, Brazil.

### Potential sources of bias

In an attempt to control potential sources of bias, the study was completed on two separate days all between 17:00 hr and 22:00 hr to standardize circadian influences^17^ in a silent room with humidity between 40% and 60% and temperature between 21 °C and 25 °C. The subjects were advised to refrain from drinking alcohol or performing exhaustive exercise 24 hours prior to the protocols and to consume caffeinated drinks only eight hours before. Subjects were recommended to wear appropriate and comfortable clothing to allow for the necessary physical effort and to eat a light meal only 2-hours before the procedures.

The descriptive profile of the individuals was defined to describe the sample, lessen the unpredictability of the variables, enhance reproducibility and physiological interpretation. We controlled age, SBP, DBP, mass (kg), height (m) and body mass index (BMI) in order to avoid impacting physiological variability.

### Initial assessment

The initial evaluation was completed to investigate the eligibility criteria and to obtain characterization information of the individuals. An anamnesis was initially performed to confirm the absence of related diseases, the use of medications, to apply a questionnaire to consider the level of physical activity, to measure BP and to examine the suitability of partaking in the experimental protocol.

The subjects were identified and categorized by collecting age, mass, height, HR, respiratory rate (RR), SBP, DBP and BMI. Levels of physical activity of the volunteers was assessed by the applying the IPAQ^14^ questionnaire, comprised of questions that assess physical activity in a typical week, which is then subdivided into activity as a means of transportation, at work, at home, relaxation, sport, exercise and leisure, and also sedentary time. Consistent with its classification, individuals can be classified as sedentary, insufficiently active, active and very active.

The anthropometric quantities were obtained according to the approvals described by Lohman *et al*.^18^. The mass was determined via a digital scale (W200/5, Welmy, Brazil) with an accuracy of 0.1 kg. Height was measured by a stadiometer (ES2020, Sanny, Brazil) with an accuracy of 0.1 cm. The BMI was calculated via the following mathematical formula: mass (kg)/height (m^2^).

### Variables, data sources and outcome measures

#### Cardiorespiratory variables

During BP measurement, the subjects remained seated; BP was verified indirectly by auscultation through a calibrated aneroid sphygmomanometer (Premium, Barueri, SP, Brazil) and stethoscope (Premium, Barueri, SP, Brazil) on the left arm^19^. To avoid misrepresentations in the quantities, the same researcher measured the cardiorespiratory parameters throughout the experiment.

The HR was evaluated by the Polar RS800cx HR monitor (Polar Electro, Finland) and RR measurement was performed by counting the respiratory incursions for one minute whilst the volunteer was unaware of the procedure to eliminate the influence of psychological stress on RR.

### HRV analysis

We followed directives from the Task Force of the European Society of Cardiology and the North American Society of Pacing and Electrophysiology^20^. Regarding HRV analysis, the HR was recorded beat-to-beat during the experimental protocol via a HR monitor (Polar RS800cx, Finland) with a sampling rate of 1 kHz. The IBI recorded were then transferred to the Polar Precision Performance Software (v. 3.0, Polar Electro, Finland) that permits visualization of HR and signal stability. Five-minute intervals were then selected and saved in a “txt” file. Next, digital filtering in the moderate mode was performed by the same Polar Precision Performance Software supplemented with manual filtering for the elimination of artifacts. For data analysis we designated stable series with 256 IBI. Only series with above 95% of sinus beats were included in the study^21^. Further details have been previously documented^22–24^.

The markers for HRV applied were the time domain analysis index of the root mean square of successive differences (rMSSD), the frequency domain index of the high frequency spectral component (HF) of the power spectral density (0.15 to 0.4 Hz) in absolute units and, the SD1 Poincaré plot (standard deviation of the instantaneous beat-to-beat variability), which signifies the dispersion of the points perpendicular to the identity line^21^.

In order to calculate the HRV indices we applied the Kubios HRV^®^ software package (Kubios^®^ HRV v.1.1, Biomedical Signal Analysis Group, Department of Applied Physics, University of Kuopio, Finland)^25^.

### Experimental protocols

After the initial assessment, the Polar RS800cx (Polar Electro, Finland) strap was placed on the subjects’ chest in the region of the distal third of the sternum. The experimental protocols were then finalized during two different days, all performed on a treadmill, with a minimum interval of 48 hours between them, such to allow for suitable physiological recovery.

The maximum effort test protocol was performed to record the maximum velocity attained by the subject. The exercise test on the treadmill (Inbramed, MASTER CI, Brazil) was performed using the Bruce incremental protocol^26^.

In the submaximal exercise protocol, the subjects performed 5 minutes initially with a speed of 6.0 km/h (+1% inclination) for ‘warming up’, followed by 25 minutes with intensity equivalent to 60% of the maximal speed reached in the maximum effort protocol with 1% of inclination^22^.

The HRV analysis was completed at the following moments: Rest (10^th^–15^th^ minute) and during recovery: M1 (5^th^–10^th^ minute), M2 (15^th^–20^th^ minute), M3 (25^th^–30^th^ minute), M4 (35^th^–40^th^ minute), M5 (45^th^–50^th^ minute) and M6 (55^th^–60^th^ minute).

**Figure 2:**
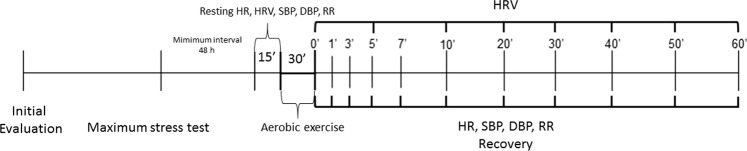
Study design.

The HR, RR, SBP, and DBP data were logged at the 15^th^ minute of the initial rest and at 1^st^, 3^rd^, 5^th^, 7^th^, 10^th^, 20^th^, 30^th^, 40^th^, 50^th^ and 60^th^ minutes after exercise (Fig. 2)^27–29^.

### Study size

The characterization of sample size was determined by the sample calculation according to a pilot protocol, wherein the online software from the website www.lee.dante.br was required based on the rMSSD index. The magnitude of the significant difference assumed was 14.11 ms, considering a standard deviation of 12.8 ms, with alpha risk of 5% and beta risk of 80%. The sample size produced, was when the smallest number was 13 individuals per group.

### Statistical analysis

For the data analysis, descriptive statistics were achieved for characterization of the sample and the results were presented with values of mean, standard deviation, minimum and maximum values.

The normality of the data was assessed via the Shapiro Wilk test. When comparing the descriptive characteristics between the groups, the unpaired Student t-test (parametric data) or the Mann-Whitney test (non-parametric data) was applied.

For comparisons of HRV indices and cardiorespiratory parameters between groups and moments (rest vs. moments of recovery), the data were initially verified for sphericity violation using the Mauchly test. The Greenhouse-Geisser correction was used when the sphericity was violated followed by analysis of mixed variance with BMI adjustment. Next, for analysis of the moments (rest vs. recovery from exercise), the covariance analysis adjusted by the BMI with Bonferroni posttest was applied since we found significant differences between groups regarding BMI.

Statistically significant differences were considered when the “*p*” value was less than 0.05 (<5%). We enforced the Minitab software (Minitab, PA, USA), GraphPad InStat - v3.06, (GraphPad Software, Inc., San Diego California USA) and IBM SPSS Statistics - v22.0 (SPSS Inc. Chicago USA).

So as to measure the magnitude of differences, the effect size was calculated through the Cohen’s *d* and Hedges’ *g*, between groups and between two time points. We assume large effect size for values greater than or equal to 0.9, medium effect size for values between 0.9 and 0.5 and lastly, small effect size for values between 0.5 and 0.25^30,31^.

Furthermore, we evaluated the association between resting SBP and HRV (at rest and during recovery from exercise). We evaluated either Pearson correlation coefficient for parametric distributions or the Spearman correlation coefficient for non-parametric distributions. We assumed strong correlations for r > 0.75 and moderate correlations for r between 0.75 and 0.5.

## Results

### Sample profile

The classification of the sample, resting SBP and DBP, the performance of the subjects in the maximal effort test and in the aerobic exercise protocol are described in Table 1. Table 1 presents the homogeneity between the groups in relation to the described variables, excepting BMI, SBP and DBP, in which there was a statistically significant difference when comparing the groups.

**Table 1:** Characterization of the sample regarding age, BMI, HRmax, Dmax, Vmax, 60%Vmax, resting SBP and DBP.

Variables	G1	G2	p value	Hedges’s g
#Age (years)	22.40 ± 3.16 [18–28]	22.55 ± 2.45 [18–28]	p = 0.7464	0.055
*BMI (Kg/m²)	21.78 ± 2.07 [18.46–24.69]	23.36 ± 1.4 [19.9–24.89]	p = 0.047	0.896
*HR max (bpm)	195.05 ± 8.70 [178–209]	196.85 ± 7.59 [184–208]	p = 0.4538	0.222
#Dmax	10.95 ± 1.82 [6.83– 16.37]	11.25 ± 1.005 [10–15.06]	p = 0.4450	0.213
*Vmax (km/h)	13.05 ± 1.87 [11–18]	13.33 ± 1.24 [12–17]	p = 0.5365	0.183
*60% Vmax (km/h)	7.83 ± 1.12 [6.6–10.8]	8 ± 0.74 [7.20–10.20]	p = 0.5365	0.183
*SBP rest (mmHg)	102.45 ± 4.11 [94–109]	114.29 ± 3.98 [110–120]	p < 0.0001	2.932
*DBP rest (mmHg)	67.85 ± 5.86 [59–82]	73.25 ± 6.20 [64–84]	p = 0.0041	0.892

Mean + Standard deviation [minimum-maximum].

Average values followed by their respective standard deviations. minimum and maximum. BMI: body mass index; HR max: maximum heart rate; Dmax: maximal distance; Vmax: maximum speed; SBP: systolic blood pressure; DBP: diastolic blood pressure; kg: kilogram; m: meters; bpm: beats per minute; km/h: kilometers/hour; mmHg: millimeters of mercury. *Unpaired Student t test. #Mann-Whitney test. G1: Individuals with SBP < 110 mmHg; G2: Individuals with SBP between 110 and 120 mmHg.

### BMI adjustment

Since we detected higher BMI in G2, we were required to perform ANCOVA with BMI adjustment. For analysis of the moments (rest vs. recovery from exercise), the covariance analysis adjusted by the BMI with Bonferroni posttest was necessary. We revealed that the data relevance was unchanged. In fact, it became more evident when removing the BMI limitation. The results acquired are presented below.

### HRV analysis

**Figure 3:**
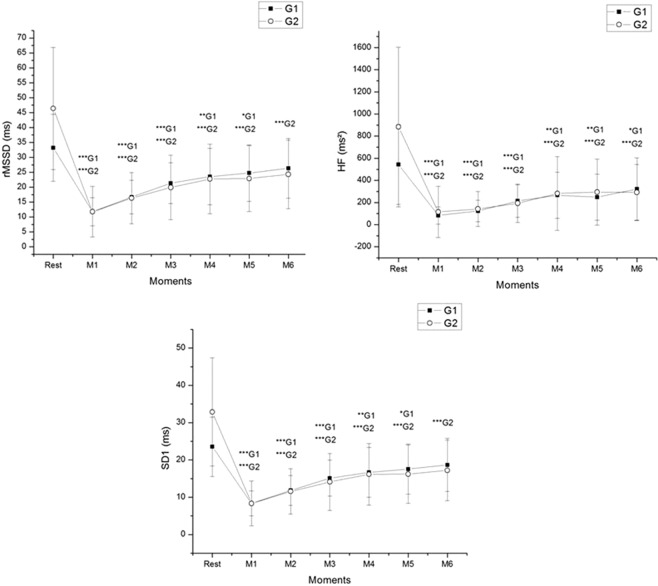
Mean values and respective standard deviations of rMSSD, HF and SD1 obtained at rest and during recovery from moderate aerobic exercise protocol. ***G1 and ***G2: Values with significant differences in relation to rest (p < 0.001); **G1: Values with significant differences in relation to rest (p < 0.01); *G1: Values with significant differences in relation to rest (p < 0.05). rMSSD: square root of the square mean of the differences between adjacent normal IBI; HF: high frequency; SD1: standard deviation of instantaneous beat-to-beat variability; ms: milliseconds; ms²: absolute units. G1: Individuals with SBP < 110 mmHg; G2: Individuals with SBP between 110 and 120 mmHg. Rest: 10^th^ to 15^th^ minute before exercise, M1: 5^th^ to 10^th^ minute, M2: 15^th^ to 20^th^ minute, M3: 25^th^ to 30^th^ minute, M4: 35^th^ to 40^th^ minutes, M5: 45^th^ to 50^th^ minute and M6: 55^th^ to 60^th^ during recovery from exercise.

Figure 3 displays the parasympathetic indexes of HRV before and during recovery from exercise in G1 and G2. There was an effect of moment for all indices examined (p = 0.001); we found interaction between moments vs groups for rMSSD (p = 0.010) and SD1 (p = 0.009), yet, there was no significance for HF index (ms²) (p = 0.066). No effects were observed between groups (rMSSD: p = 0.165; HF: p = 0.05; SD1: p = 0.165).

Regarding the rMSSD index, we observed that it recovered after 50 minutes following exercise in G1, whereas it did not recover until 60 minutes after exercise in G2. The HF index (ms²) was not recovered until 60 minutes after exercise in G1 and G2. The SD1 index recovered after 50 minutes following exercise in G1, but it did not recover until 60 minutes after exercise in G2 (Fig. 3).

In G1, we observed large effect size for all HRV indices between rest vs. M1, rest vs. M2, rest vs. M3 and rest vs. M4. We detected a large effect size for HF and medium effect size for rMSSD and SD1 between rest vs. M5. Medium effect size was observed for all HRV indices between rest vs. M6.

We detected in G2 large effect size for all HRV indices between rest vs. all moments.

### Cardiorespiratory variables

**Figure 4:**
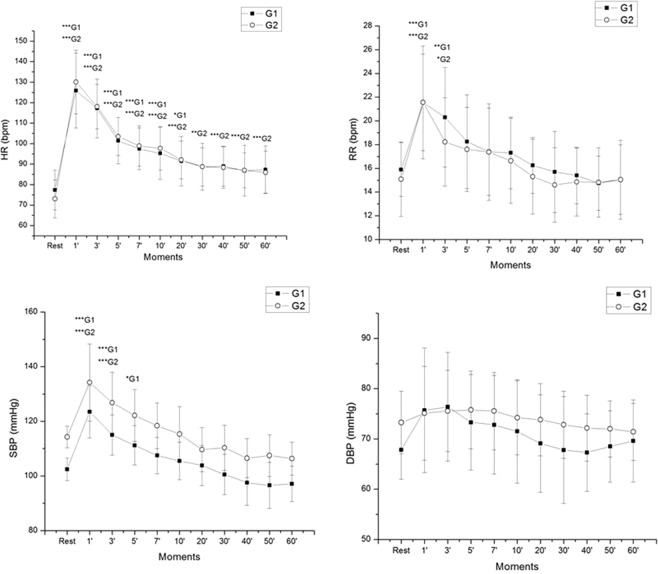
Mean values and respective standard deviations of HR, RR, SBP and DBP obtained at rest and during recovery from moderate aerobic exercise protocol. ***G1 and ***G2: Values with significant differences in relation to rest (p < 0.001); **G1 and **G2: Values with significant differences in relation to rest (p < 0.01); *G1 and *G2: Values with significant differences in relation to rest (p < 0.05). SBP: systolic blood pressure; DBP: diastolic blood pressure; HR: heart rate; RR: respiratory rate; mmHg: millimeters of mercury; bpm: beats per minute; rpm: breaths per minute. G1: Individuals with SBP < 110 mmHg; G2: Individuals with SBP between 110 and 120 mmHg. Rest: 10^th^ to 15^th^ minute before exercise, M1: 5^th^ to 10^th^ minute, M2: 15^th^ to 20^th^ minute, M3: 25^th^ to 30^th^ minute, M4: 35^th^ to 40^th^ minutes, M5: 45^th^ to 50^th^ minute and M6: 55^th^ to 60^th^ during recovery from exercise.

Cardiorespiratory variables at rest and during recovery from exercise are available in Fig. 4. We observed effect of moment for HR, RR and SBP (p < 0.001). There was no interaction between groups (HR: p = 0.924, RR: p = 0.935, SBP: p = 0.898, DBP: p = 0.61). We detected a group effect for RR, SBP and DBP (p < 0.001), but there was no group effect for HR (p = 0.866).

Concerning HR recovery, a statistically significant difference was observed at recovery times up to the 20^th^ minute for G1 and up to 60^th^ minute for G2 compared to rest. Regarding RR, a statistically significant difference was found until the 3^rd^ minute of recovery compared to rest for both groups (Fig. 4).

Concerning SBP, we observed a statistically significant difference in recovery moments up to the 5^th^ minute for G1 and up to the 3^rd^ minute for G2 compared to rest. For DBP, we attained no significant difference between rest and recovery in either group.

In G1, we detected large effect size for HR between rest vs. 1′, rest vs. 3′, rest vs. 5′, rest vs. 7′, rest vs. 10′, rest vs. 20′, rest vs. 30′, rest vs. 40′ and rest vs. 60′. Medium effect size for HR was achieved between rest vs. 50′. Regarding RR, there was large effect size between rest vs. 1′ and rest vs. 3′ and medium effect size between rest vs. 5′, rest vs. 10′ and rest vs. 50′. Large effect size for SBP was reported between rest vs. 1′, rest vs. 3′, rest vs. 5′, rest vs. 7′, rest vs. 40′, rest vs. 50′ and rest vs. 60′. Concerning DBP, we detected large effect size between rest vs. 3′ and medium effect size between rest vs. 1′, rest vs. 5′ and rest vs. 7′.

In regard to G2, we revealed large effect size between rest vs. all moments for HR. There was large effect size between rest vs. 1′ and rest vs. 3′ and medium effect size between rest vs. 5′ and rest vs. 7′ for RR. On SBP, we observed large effect size between rest vs. 1′, rest vs. 3′, rest vs. 5′, rest vs. 40′, rest vs. 50′ and rest vs. 60′ and medium effect size between rest vs. 7′, rest vs. 20 and rest vs. 30′.

### Association between resting SBP and HRV

According to Table 2, we detected no significant correlation between resting SBP and HRV at rest and during recovery from exercise.

**Table 2:** Correlation between resting SBP and HRV indices.

	Rest		M1		M2		M3		M4		M5		M6	
	r	p	r	p	r	p	r	p	r	p	r	p	r	p
rMSSD	0.29	0.11	0.15	0.42	0.05	0.77	−0.09	0.59	−0.01	0.94	−0.07	0.68	−0.09	0.95
HF	0.21	0.25	0.28	0.12	0.19	0.29	0.11	0.54	0.06	0.74	0.21	0.26	0.07	0.68
SD1	0.28	0.11	0.15	0.42	0.06	0.79	−0.08	0.6	−0.02	0.93	−0.07	0.68	−0.1	0.96

Legend: rMSSD: square root of the square mean of the differences between adjacent normal IBI; SD1: standard deviation of instantaneous beat-to-beat variability; HF: high frequency; G1: Individuals with SBP < 110 mmHg; G2: Individuals with SBP between 110 and 120 mmHg; Rest: 10th to 15th minute before exercise, M1: 5th to 10th minute, M2: 15^th^ to 20^th^ minute, M3: 25^th^ to 30^th^ minute, M4: 35^th^ to 40^th^ minutes, M5: 45^th^ to 50^th^ minute and M6: 55^th^ to 60^th^ during recovery from exercise.

## Discussion

Pre-hypertension has been previously documented as a significant risk factor for cardiovascular disorders^8^. With the purpose of providing information to confirm whether SBP below 110 mmHg is better than SBP between 110 mmHg and 120 mmHg, we evaluated cardiorespiratory and autonomic recovery following submaximal aerobic exercise in normotensive individuals with different SBP values. As a significant finding, we discovered that normotensive subjects’ higher SBP presented delayed recovery following moderate exercise compared to individuals with lower SBP by examining HR and HRV. Instead, there were no differences between groups regarding recovery of RR and blood pressure. Similarly, we attempted to evaluate the association between resting SBP and HRV during recovery from exercise. Yet, we did not observe significant interaction between the stated variables.

Both groups presented normal physiological responses to exercise, because all subjects were physically active and healthy. Under normal conditions during exercise, vasodilatation in the active skeletal muscles and increased vessel resistance in less active organs are vital to supply the metabolic demand. There is an increase in cardiac output to secure blood flow, elevating HR and SBP and, DBP changes slightly during exercise. After exercise cessation, a temporary decline in SBP below pre-exercise levels is as a result of peripheral vasodilation^32^.

HR during exercise is controlled by neural and peripheral mechanisms (mechanoreceptors and/or metaboreceptors)^29,30^. Muscle metaboreceptors are activated in response to accumulation of metabolites (H+, K+, adenosine, lactate), decreasing baroreflex sensitivity^33–36^. These responses contribute to reduced vagal activity and increased sympathetic activity during exercise, resulting in elevated HR. Closely after exercise cessation, there is a rapid decline in HR attributable to vagal reactivation^33–36^.

Peçanha *et al*.^35^ recommended that progressive removal of metabolites during exercise recovery decreases metaboreflex activation, restoring baroreflex activity. Also, it induces enlarged vagal activity and decreased sympathetic activity, leading to a progressive decrease in HR. Together, we may recommend that this mechanism is faster in subjects with SBP < 100 mmHg compared to individuals with SBP between 110 and 120 mmHg.

In a preceding study^36^, hypertensive and normotensive subjects (SBP approximately 110–120 mmHg and DBP almost 75–80 mmHg) performed exercise sessions on a cycle ergometer (30 minutes, 70% of peak VO_2_) and HR recovery was evaluated. The researchers’ reported delayed autonomic recovery in hypertensive subjects.

Thus, it is worth highlighting that autonomic recovery after exercise has been associated with cardiovascular risk, specifically the slower the autonomic recovery, the higher the cardiovascular risk^12,13^. So, we conjectured in our study that higher resting SBP in normotensive subjects would be related to delayed HRV recovery from exercise.

Our results regarding HR and HRV analysis during recovery from exercise support this proposal, since autonomic recovery was slower in subjects with resting SBP between 110 and 120 mmHg compared to individuals with resting SBP below 110 mmHg.

Consistent with our data, RR was analogous between the two groups. During exercise in normal conditions, neurogenic stimuli from the cerebral cortex and limbs exercising result in an early and swift increase in respiration. After a short-term plateau, minute ventilation gradually increases to a stable level that adequately meets the demands for metabolic gas exchange. Upon cessation, there is a gradual return to the initial resting state^37^.

Additionally, SBP and DBP recovery following exercise were unchanged between groups. Consequently, we reinforce that all subjects presented normal physiological responses within the normal limits of the cardiorespiratory variables. We anticipated these responses since all subjects were physically active and healthy.

Concerning HRV, normotensive individuals with higher resting SBP presented delayed vagal recovery from exercise compared to normotensive subjects with lower resting SBP. An earlier study^38^ with normotensive (n = 32), pre-hypertensive (n = 28) and hypertensive (n = 31) individuals detected that the autonomic imbalance in pre-hypertensive subjects was owing to the proportional increase of sympathetic activity and to vagal inhibition. These authors highlighted that rMSSD was reduced and the LF/HF ratio was increased in the hypertensive group.

Since normotensive subjects with higher resting SBP presented delayed HR and HRV recovery following exercise, we advocate that resting SBP below 110 mmHg is clinically advantageous rather than resting SBP between 110–120 mmHg. Some physiological mechanism may be wished-for to clarify this finding. Hypertension is related to increased oxidative stress, vascular aging, inflammation^39^ and changes in brainstem areas regulating autonomic function^40^. We anticipate that the abovementioned physio-pathological factors are more intense in normotensive subjects with higher resting SBP compared to individuals with lower resting SBP.

Some areas in our study, require highlighting. We investigated a small sample size; so, we support the need to reproduce these findings in larger samples. Although we excluded individuals with BMI above 25 kg/m^2^, the G2 presented higher BMI than the G1. Considering this, we applied the mixed variance analysis adjusted by the BMI and the covariance analysis adjusted by the BMI. We documented that the data relevance was not influenced by the inter group BMI difference. In actual fact, our results were more evident when eliminating the BMI.

We did not evaluate the low frequency (LF) index of the spectral analysis nor the LF/HF ratio as it has been reported to be empirically unsupported and theoretically flawed. The most serious concern is that LF does not index sympathetic activity and, so, there is a lack of rationale and compelling evidence that its strength in relation to the HF component would index relative strength of vagal and sympathetic signaling^41,42^. The pNN50 (percentage of adjacent IBI with a difference of duration greater than 50 ms) was not evaluated because the Task Force of the European Society of Cardiology and the North American Society of Pacing and Electrophysiology^5,20^ specified that the rMSSD provided better statistical properties. The SDNN (standard deviation of all normal IBI recorded in a time interval) was not evaluated because it is more reliable for long term analysis^43^, not in the short-term; as here.

We evaluated only young males so as to constitute a standardized population based on similar gender, health and physical status. There is a transformation between age, physical conditioning, gender and influence of sex hormones on autonomic and cardiovascular function^44^. Blood pressure is typically lower in young women compared to young men and rates of hypertension are lower in young women compared to young men^45^. The role of β‐adrenergic receptors was implicated to explain the differences between young women and young men concerning vasoconstriction responses and hypertension development. Likewise, aging is related to deteriorated global autonomic regulation and decreased HRV^46^. The arterial blood pressure autonomic activity is greater in older women compared to young women. The sympathetic nerve activity is enlarged in older women, elevating the incidence of hypertension in older women^47^. As a consequence, we are unable to extrapolate our data to subjects with different genders and age groups. Our results offer challenges for future studies to better investigate different populations.

Our data provided recommendations that support resting SBP < 110 mmHg as clinically advantageous than resting SBP between 110 and 120 mmHg. These results draw attention to preventive strategies, to facilitate avoiding the risky control of the BP levels (worldwide); cooperating clinically to reduce cardiovascular morbidity and mortality.

## Conclusion

Healthy normotensive subjects with higher resting SBP exhibited slower autonomic recovery from moderate exercise compared to individuals with lower resting SBP. Nevertheless, no differences were detected for blood pressure and RR. Our results recommend that resting SBP below 110 mmHg may be clinically advantageous than SBP between 110–120 mmHg, suggesting a reduced probability of developing cardiovascular disease in subjects with lower SBP. We highlight this outcome for medical clinicians working worldwide to achieve beneficial and preventive strategies.

## Supplementary information

Supplementary file 1: Effect size through Cohen’s d for HRV. Supplementary file 2: Effect size through Cohen’s d for cardiorespiratory variables.
