# Supplementary material for: Lower Systolic Blood Pressure in Normotensive Subjects is Related to Better Autonomic Recovery Following Exercise
Source: Sci Rep. 2020 Jan 22;10:1006. doi: 10.1038/s41598-020-58031-5 (PMC6976706; doi:10.1038/s41598-020-58031-5)
Supplement: Supplementary file 1 — Effect size through Cohen’s d for HRV. Supplementary file 2: Effect size through Cohen’s d for cardiorespiratory variables. [file 41598_2020_58031_MOESM1_ESM.docx]

**LOWER SYSTOLIC BLOOD PRESSURE IN NORMOTENSIVE SUBJECTS IS RELATED TO BETTER AUTONOMIC RECOVERY FOLLOWING EXERCISE**

Letícia Santana de Oliveira^1^, Anne Michelli G. G. Fontes^1^, Ana Laura Ricci Vitor^1^, Franciele M. Vanderlei^1^, David M. Garner^2^, Vitor E. Valenti^1, 3*^

^1^Post-Graduate Program in Physical Therapy, São Paulo State University, UNESP, Presidente Prudente, SP, Brazil. ^2^Cardiorespiratory Research Group, Department of Biological and Medical Sciences, Faculty of Health and Life Sciences, Oxford Brookes University, Headington Campus, Gipsy Lane, Oxford, OX3 0BP, United Kingdom. ^3^Autonomic Nervous System Center (CESNA), São Paulo State University, UNESP, Marilia, SP, Brazil.

***Correspondence to:** Vitor E. Valenti

UNESP, Av. Hygino Muzzi Filho, 737. Mirante

17.525-900 - Marilia, SP. Brazil

Phone: +55 14 3402-1300

E-mail: vitor.valenti@unesp.br

**Supplementary file 1:** Effect size through Cohen’s *d* for HRV.

| **G1** | **Rest vs. M1** | **Rest vs. M2** | **Rest vs. M3** | **rest vs. M4** | **Rest vs. M5** | **Rest vs. M6** |
| --- | --- | --- | --- | --- | --- | --- |
| **rMSSD** | 2.496 | 1.862 | 1.278 | 0.933 | 0.817 | 0.649 |
| **HF** | 1.763 | 1.588 | 1.196 | 0.94 | 1.002 | 0.683 |
| **SD1** | 2.495 | 1.859 | 1.277 | 0.932 | 0.815 | 0.648 |
| **G2** | **Rest vs. M1** | **Rest vs. M2** | **Rest vs. M3** | **Rest vs. M4** | **Rest vs. M5** | **Rest vs. M6** |
| **rMSSD** | 2.206 | 1.914 | 1.616 | 1.416 | 1.428 | 1.33 |
| **HF** | 1.434 | 1.42 | 1.315 | 1.07 | 1.067 | 1.094 |
| **SD1** | 2.208 | 1.915 | 1.617 | 1.416 | 1.43 | 1.332 |

**Legend:** rMSSD: square root of the square mean of the differences between adjacent normal IBI; HF: high frequency; SD1: standard deviation of instantaneous beat-to-beat variability. G1: Individuals with SBP<110 mmHg; G2: Individuals with SBP between 110 and 120 mmHg. Rest: 10^th^ to 15^th^ minute before exercise, M1: 5^th^ to 10^th^ minute, M2: 15^th^ to 20^th^ minute, M3: 25^th^ to 30^th^ minute, M4: 35^th^ to 40^th^ minutes, M5: 45^th^ to 50^th^ minute and M6: 55^th^ to 60^th^ during recovery from exercise.

**Supplementary file 2:** Effect size through Cohen’s *d* for cardiorespiratory variables.

| **G1** | **Rest vs. 1’** | **Rest vs. 3’** | **Rest vs. 5’** | **Rest vs. 7’** | **Rest vs. 10’** | **Rest vs. 20’** | **Rest vs. 30’** | **Rest vs. 40’** | **Rest vs. 50’** | **Rest vs. 60’** |
| --- | --- | --- | --- | --- | --- | --- | --- | --- | --- | --- |
| **HR** | 3.309 | 3.245 | 2.282 | 2.015 | 1.577 | 1.284 | 1.07 | 1.189 | 0.844 | 0.934 |
| **RR** | 1.714 | 1.304 | 0.73 | 0.491 | 0.525 | 0.151 | 0.068 | 0.215 | 0.504 | 0.324 |
| **SBP** | 2.845 | 2.1 | 1.491 | 0.91 | 0.539 | 0.235 | 0.318 | 0.745 | 0.892 | 0.986 |
| **DBP** | 0.809 | 0.98 | 0.689 | 0.612 | 0.435 | 0.155 | 0.005 | 0.08 | 0.099 | 0.246 |
| **G2** | **Rest vs. 1’** | **Rest vs. 3’** | **Rest vs. 5’** | **Rest vs. 7’** | **Rest vs. 10’** | **Rest vs. 20’** | **Rest vs. 30’** | **Rest vs. 40’** | **Rest vs. 50’** | **Rest vs. 60’** |
| **HR** | 4.467 | 4.465 | 3.282 | 2.709 | 2.471 | 2.051 | 1.682 | 1.565 | 1.562 | 1.314 |
| **RR** | 1.61 | 0.914 | 0.751 | 0.632 | 0.461 | 0.07 | 0.153 | 0.073 | 0.085 | 0.011 |
| **SBP** | 1.921 | 1.493 | 1.078 | 0.622 | 0.134 | 0.731 | 0.613 | 1.342 | 1.116 | 1.546 |
| **DBP** | 0.233 | 0.318 | 0.358 | 0.328 | 0.141 | 0.082 | 0.069 | 0.173 | 0.213 | 0.311 |

**Legend:** rMSSD: square root of the square mean of the differences between adjacent normal IBI; HF: high frequency; SD1: standard deviation of instantaneous beat-to-beat variability. G1: Individuals with SBP<110 mmHg; G2: Individuals with SBP between 110 and 120 mmHg; Rest: 10^th^ to 15^th^ minute before exercise, M1: 5^th^ to 10^th^ minute, M2: 15^th^ to 20^th^ minute, M3: 25^th^ to 30^th^ minute, M4: 35^th^ to 40^th^ minutes, M5: 45^th^ to 50^th^ minute and M6: 55^th^ to 60^th^ during recovery from exercise.
